# In Silico and In Vivo Evaluation of microRNA-181c-5p’s Role in Hepatocellular Carcinoma

**DOI:** 10.3390/genes13122343

**Published:** 2022-12-12

**Authors:** Omnia Nasser Abd ElAziz, Asmaa M. Elfiky, Mohamed A. Yassin, Fatma El-Zahraa Abd El-Hakam, Eman M. Saleh, Mahmoud El-Hefnawi, Rania Hassan Mohamed

**Affiliations:** 1 Department of Biochemistry, Faculty of Science, Ain Shams University, Cairo 11566, Egypt; 2 National Research Centre, Environmental and Occupational Medicine Department, Environmental Research Division, Cairo 12622, Egypt; 3 Center of Excellence, National Research Centre, Advanced Materials and Nanotechnology Laboratory, Cairo 12622, Egypt; 4 National Research Centre, Packaging Materials Department, Cairo 12622, Egypt; 5 Department of Pharmacology, Faculty of Medicine for Girls, Al-Azhar University, Cairo 11884, Egypt; 6 National Research Centre, Biomedical Informatics and Cheminformatics Group, Informatics and Systems Department, Cairo 12622, Egypt

**Keywords:** hepatocellular carcinoma, nano-delivery, miR-181c-5p, Fbxl3, oncogenic miRNA

## Abstract

Hepatocellular carcinoma (HCC) is a fatal disease, accounting for 75–85% of primary liver cancers. The conclusive research on miR-181c-5p’s role in hepatocarcinogenesis, whether it has oncogenic effects or acts as a tumor repressor, is limited and fluctuating. Therefore, the current study aimed to elucidate the role of miR-181c-5p in HCC in silico and in vivo. The bioinformatics analysis of miR-181c-5p expression data in HCC using several databases strongly shed light on its involvement in HCC development, but also confirmed the fluctuating data around its role. miR-181c-5p was proven here to have an oncogenic role by increasing HepG2 cells’ viability as confirmed by MTT analysis. In addition, miR-181c-5p was upregulated in the HCC positive control group and progressed the HCC development and malignant features by its forced expression in an HCC mouse model by targeted delivery using a LA-PAMAM polyplex. This is indicated by the cancerous gross and histological features, and the significant increase in liver function biomarkers. The functional enrichment bioinformatics analyses of miR-181c-5p-downregulated targets in HCC indicated that miR-181c-5p targets were significantly enriched in multiple pathways and biological processes involved in HCC development. Fbxl3, an example for miR-181c-5p potential targets, downregulation and its correlation with miR-181c-5p were validated by qPCR. In conclusion, miR-181c-5p is upregulated in HCC and has an oncogenic role enhancing HCC progression.

## 1. Introduction

Hepatocellular carcinoma (HCC) is a lethal disease; it is the fifth most frequent cancer [1] and the fourth largest contributor to cancer deaths worldwide [2]. It represents the fourth most widespread cancer in Egypt [3,4]. The most remarkable risk factors for the emergence of HCC are chronic liver diseases and cirrhosis [5]. Recognizing the molecular pathogenesis of HCC is insufficient and inconclusive so far. Consequently, to develop an efficient therapeutic strategy, conducting a more inclusive analysis of HCC is demand [6]. Lately, studies focused on targeted therapies, due to the aggressiveness of the tumor nature [7].

Microarray expression data suggested that microRNA (miRNA)-altered expression is ordinary in cancer [8,9]. MiRNAs are short non-coding RNAs consisting of 20–24 nucleotides in sequence and can affect various processes related to cancer onset and progression. These non-coding RNAs could function as posttranscriptional regulators by targeting mRNAs [10,11,12]. Oncogenic miRNA upregulation enhances tumorigenesis by restricting tumor suppressor mRNAs’ translation (e.g., miR-125, miR125b, and miR17-22) [13,14]. On the contrary, tumor suppressor miRNAs’ expression is declined in various cancer types and usually prevent the development of tumors by inhibiting oncogenes (e.g., miR-145, miR133a, miR-143, miR-195, and miR-34a) [15,16,17,18].

Multiple biological activities, such as immune response, mitochondrial function, autophagy, apoptosis, and cell proliferation can be regulated by the miR-181 family [19]. In humans and mice, the miR-181 family includes four members (miR-181a, miR-181b, miR-181c, and miR-181d). Three distinct transcripts encode the miR-181 family, which is located on three distinct chromosomes [20]. Members of the miR-181 family play a crucial role in cancer by acting as oncogenic or tumor suppressor miRNAs [21]. The downregulation of miR-181a, b, and d decreased the migration of hepatic cancer cells, showing that the family of miR-181 has a vital role in the development of liver cancer. [22]. Various investigations have reported that miR-181c had abnormal expression in various tumors [23]. MiR-181c was downregulated and functioned as a tumor suppressor in metastatic neuroblastoma (NB) [24], breast cancer [25], glioblastoma [26], oral cancers [27], and gastric cancer [28]. In contrast, miR-181c acts as a robust oncomiR in inflammatory breast cancer (IBC) [29] and pancreatic cancer [30]. The same scenario was addressed in HCC, while J. Ji et al. (2009) and Indrieri et al. (2020) demonstrated that the miR-181 mature family members (miR-181a, miR-181b, miR-181c, miR-181d) were increased significantly in HCC patients, HCC stem cells, and progenitors [21,31], other studies demonstrated that miR-181c was downregulated [32,33], and inhibited HCC growth and metastasis [34].

Nanoparticles (NPs) exhibited remarkable properties concerning gene delivery when used for in vivo therapy. It can target and penetrate certain tissues or cells due to their smaller size, in addition to their lower vulnerability to clearance via the reticuloendothelial system [18,35,36]. Polyamidoamine (PAMAM) was studied extensively for nucleic acids’ (NAs) delivery. PAMAM polymers can be manufactured in a defined structure, at which the high density of its cationic charges exhibits electrostatic interactions with NAs as a proton sponge. It can condense polyanionic NAs, construct dendriplexes to prevent the degradation of the NAs, enable cellular uptake on varied cell lines, and allow endo-lysosomes’ localization, due to the “proton sponge” effect which favors endosomal escape [37,38]. A ligand called Lactobionic acid (LA) has a galactosyl moiety that binds to the asialoglycoprotein receptor (ASGP-R) that is overexpressed in HCC to permit efficient targeted delivery to HCC cells [39,40,41]. The LA-modified delivery system could improve drug or gene uptake into hepatoma cells [42]. We previously used the LA conjugated hyperbranched PAMAM for efficient therapeutic delivery of miR-218 to the HepG2 cell line, and HCC mice tissue [43].

Since HCC implicated an elevated risk of recurrence and poor prognosis, finding more details about its molecular pathogenesis is very demanding. Due to the fluctuating data about the role of miR-181c-5p in HCC, identification of the role of miR-181c-5p in HCC may provide more clues for HCC therapeutic strategies. In this study, we aimed to investigate the role of miR-181c-5p in HCC in vitro and in vivo. Moreover, the molecular mechanisms of miR-181c-5p were also investigated by bioinformatics functional enrichment analysis of miR-181c-5p targets in HCC.

## 2. Materials and Methods

### 2.1. Database Analysis of the Expression of miR-181c-5p

The human miRNA tissue atlas is available at (https://ccb-web.cs.uni-saarland.de/tissueatlas/) (accessed on 2 October 2021) and was used to demonstrate miR-181c-5p organ specificity, as miRNA research showed distinct sets of miRNAs that expressed in varied tissues and types of cells. Using dbDEMC2 software (http://www.picb.ac.cn/dbDEMC) (accessed on 4 October 2021), the expression status of miR-181c-5p in normal and tumor tissues, including HCC, was determined. An integrated database called DbDEMC (database of Differentially Expressed MiRNAs in Human Cancers) holds high throughput data on how miRNAs are expressed differentially in human cancers [44]. Other miRNA disease association databases, HMDD [45], miR2Disease [46], and miRcancer [47], that are widely used in the literature were utilized to clarify the differential expression status of miR-181c-5p in HCC. StarBase v2.0 (http://starbase.sysu.edu.cn/) (accessed on 4 October 2021) was used to perform the survival analyses for miR-181c-5p sourced from TCGA [48].

### 2.2. Materials and Cells

From Merck, we obtained the chemicals Diethylnitrosamine (DEN) and Carbon tetrachloride (CCl4). Cell Biolabs in the USA provided the pEGP-miR cloning and expression vector, as well as the pEGP-miR null control vector. The Shanghai Cell Institute Country Cell Bank (Shanghai, China) provided the human HCC cell lines HepG2 and MTT (3-(4,5-dimethylthiazol-2-yl)-2,5-diphenyltetrazolium bromide) (Sigma-Aldrich, St. Louis, MO, USA). HepG2 cell lines were preserved in Dulbecco’s Modified Eagle Medium (DMEM) with Ampicillin/Streptomycin (Lonza, Basel, Switzerland), 10% fetal bovine serum (FBS), and 10 g/L L-glutamine with 4.5 g/L glucose (Lonza, Basel, Switzerland). Inside a humid incubator with 5% CO_2_ at 37 °C, cells were preserved.

### 2.3. Recombinant pmiR-181c-5p Construct Synthesis

The sequence of pri-miR-181c-5p was amplified from the genomic DNA of human Peripheral Blood Mononuclear Cells (PBMCs), extracted by QIAamp genomic DNA kit (QIAGEN, USA), using Thermo Scientific DreamTaq Green PCR master mix (Thermo Fisher Scientific, Waltham, MA, USA) and forward primer: 5′-TCGA-GGATCC- ACTTAAGGAGCGGGCTTGAG-3′ and reverse primer: 5′-TCGA-GCTAGC- TCACAACCCACCGACAACA-3′, according to the manufacturer’s instruction. The PCR for pri-miR-181c-5p amplification was carried out as follows: 95 °C for 5 min; and 35 cycles of 94 °C for 15 s, 54 °C for 30 s, and 72 °C for 30 s. The amplified product was subsequently cloned in a pEGP-miR vector to form pmiR-181c-5p with the aid of Thermo Scientific T4 DNA ligase (Thermo Fisher Scientific, Waltham, MA, USA). The cloned constructs were transformed subsequently into the cells of Escherichia coli TOP10 and confirmed by sequencing. Following the manufacturer’s guidelines, the verified clone was purified by an endotoxin-free GeneJET Plasmid Maxiprep Kit from Thermo Scientific (Thermo Fisher Scientific, Waltham, MA, USA). The Thermo Fisher Scientific Nanodrop 2000 spectrophotometer (Thermo Fisher Scientific, Waltham, MA, USA) was used to measure the concentration and purity of the pmiR-181c-5p construct. The pEGP-miR null vector (pNull) was considered a control vector.

### 2.4. Polyplex Formulation of pEGP-miR-181c-5p

The LA-PAMAM-pmiR-181c-5p and LA-PAMAM-pNull polyplexes were prepared directly before usage, according to the previous method [43] by diluting LA-PAMAM and pmiR-181c-5p or pNull separately in phosphate buffer saline (PBS; pH 7.4) to accomplish equal volumes of the required concentrations. Then, both solutions were set to equilibrate at room temperature for five minutes. The LA-PAMAM solution was added to the pmiR-181c-5p or pNull solution and vortexed gently for 5 s. The weight ratio of LA-PAMAM to plasmid was 2:1. The formed polyplexes of LA-PAMAM-pmiR-181c-5p and LA-PAMAM-pNull afterward were incubated at room temperature for 30 min to be ready to use.

### 2.5. LA-PAMAM-pmiR-181c-5p Transfection

In 8-well chamber slides from Thermo Scientific (Thermo Fisher Scientific, Waltham, MA, USA), 2 × 10^4^ HepG2 cells were seeded in each well the day before transfection for overnight culture. A volume of 20 μL LA-PAMAM-pmiR-181c-5p, (0.2 μg pmiR-181c-5p in a ratio of 2:1 LA-PAMAM: pmiR-181c-5p) was added up to 180 μL of pre-warmed DMEM media to prepare the transfection mixture [43]. After that, the seeded HepG2 cells were transfected by the diluted transfection mixture of the LA-PAMAM-pmiR-181c-5p. For 48 h, the transfected and untransfected (control) HepG2 cells were permitted to grow in 37 °C and 5% CO_2_. The cells were assessed by green fluorescent protein (GFP) screening expression in comparison to untransfected cells by a fluorescence Zeiss Axio imager Z2 microscope (Zeiss, Goettingen, Germany). Additionally, 10^5^ cells/well of HepG2 cells in 6-well plates were seeded and the cells were cultured for 24 h at 37 °C with 5% CO_2_ in a humidified atmosphere. Then, 4 μg plasmid DNA of LA-PAMAM-pNull or LA-PAMAM-pmiR-181c-5p, in a 2:1 ratio LA-PAMAM: plasmid was added. Forty-eight hours post-transfection, cells were harvested for qPCR analysis.

### 2.6. Cell Viability Study

The MTT colorimetric assay (3-(4,5-dimethylthiazol-2-yl)-2,5-diphenyltetrazolium bromide) was performed to evaluate the viability of the cells after treatment with various concentrations of LA-PAMAM-pmiR-181c-5p. In 96-well plates, 2 × 10^4^ HepG2 cells per well were seeded and then maintained for 24 h at 37 °C with 5% CO_2_ in a humidified environment. The transfection mixture was prepared by adding LA-PAMAM-pNull or LA-PAMAM-pmiR-181c-5p (20 μL) (in different concentrations {0.5–2 μg} plasmid DNA {the ratio of LA-PAMAM to plasmid was 2:1}) to the pre-warmed media (180 μL). This mixture was let to sit for 5 min at room temperature. Afterward, the HepG2 cells were transfected with the transfection mixture (200 μL) directly and incubated for 48 h. After transfection, 40 μL of 5 g/L MTT per well was added and incubated for 4 h at 37 ° C with cells, then the media was discarded and the acidified isopropanol solution was added. Assessment of cell viability by measuring the absorbance value for each well at 570 nm on a microplate spectrophotometer was carried out.

### 2.7. Animal Experiment

**Figure 1:**
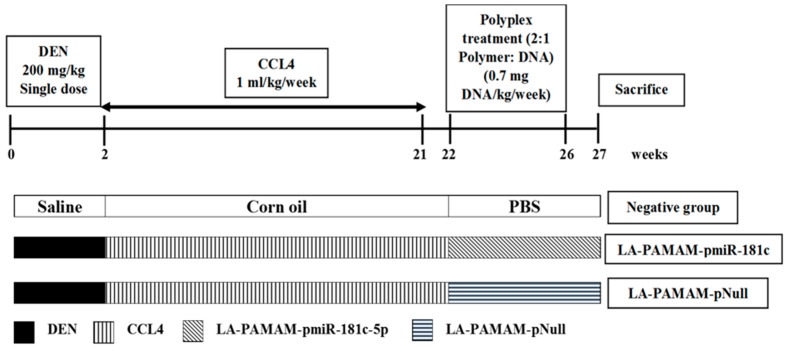
Schematic diagram of experimental animal design.

The experiments on animals were carried out as stated by the guidelines of the National Research Center Animal Care Committee and approved according to ethics (Approval Number 10431). Male Balb-c mice (8 weeks in age) (body weights 24 g ± 4) (*n* = 80) were retained in a temperature-controlled atmosphere (12 h light/dark cycle at 24 °C, drinking water and feed ad libitum). Mice were adapted to the laboratory environment one week before the experiment’s start. Mice were divided randomly into 2 groups: the negative control group (*n* = 20) and the HCC group (*n* = 60). HCC induction and treatment were performed based on the modified protocol from Salah et al. (2019). Briefly, liver cancer was induced by intraperitoneal (i.p.) injection of mice by a single dose of DEN (freshly diluted in sodium chloride saline solution with a sterility level of 0.9%), followed by 20 doses of CCl4 (corn oil dissolved 1:2 *v*/*v*) received by oral gavage (once/week). HCC was confirmed in the HCC group by liver gross examination and histopathological investigation until the prevalence of liver tumors is expected to become 100% (Supplementary Figure S1). Then, the group of HCC was split into two subgroups treated with DNA-polyplexes, into a LA-PAMAM-pmiR-181c-5p treated group (*n* = 30), and a LA-PAMAM-pNull treated group (*n* = 30; as HCC positive control). The polyplexes treated groups were injected intravenously (i.v.) via tail vein with DNA-polyplexes in a ratio 2:1 polymer: DNA for five doses following Salah et al. 2019. A single i.p. dose of saline given to the negative control group and after 2 weeks corn oil was received by oral gavage for 20 weeks, then PBS for the following 5 weeks. The detailed timeline and doses of treatment are shown in Figure 1. Ultimately, 7 days following the last injection, the mice were sacrificed.

### 2.8. Blood and Tissue Sampling

Body weight Change (BWC) % was calculated by recording the body weight of all mice at the experiment’s beginning and end. The liver tissue organs were collected, washed with PBS, and weighed to obtain the relative liver weight (RLW). BWC and RLW were calculated for every single mouse. Hepatic tissue specimens were collected and liver tumors were identified during sacrifice by macroscopic examination of the liver. Then, the liver tissue was prepared for histopathological examinations. For further biochemical and molecular analysis, blood samples were collected from each mouse group and hepatic tissue specimens were excised and directly frozen at −80 °C.

### 2.9. Biochemical Analysis

Colorimetric assays (BIOLABS, Paris, France) were used to measure the serum enzymes aspartate aminotransferase (AST) and alanine aminotransferase (ALT). Using a mouse α-fetoprotein ELISA kit from Elabscience Biotechnology Co., following the directions from the manufacturer (Houston, TX, USA), α-fetoprotein (AFP) was assessed.

### 2.10. Histopathological Analysis

Hepatic tissue specimens (including grossly visible tumors) were excised in sections of 3 to 5 mm thick, followed by fixation in neutral buffered formalin 10%. The hepatic tissue specimens were paraffin wax embedded, sections were cut at a thickness of 5 μm and hematoxylin and eosin (H&E) stained, following the previous method [49]. The sections were examined using an Olympus digital camera mounted on an Olympus microscope with a 1/2× power adaptor. The 4-scale Edmondson and Steiner system was used to classify HCC lesions [50].

### 2.11. Prediction of the Targets of miR-181c-5p

MiR-181c-5p’s sequence and expression pattern was obtained using the miRBase database (http://www.miRbase.org/) (accessed on 8 October 2021) and phenomiR database (http://mips.helmholtz-muenchen.de/phenomiR/) (accessed on 8 October 2021), respectively. To predict a miR-181c-5p target, the miRWalk v2.0 database was used [51]. The MiRWalk server offers the targets of miRNA that have been generated by the intersection of various prediction algorithms. In the current analysis, the following algorithmic target prediction was chosen: miRWalk, TargetScan v7.0, MiRmap, MiRanda, Pictar, and RNA22 to attain the common predicted targets with a cut-off *p*-value < 0.05. We selected only those target genes downregulated in HCC to pick the predicted target gene of the present study [52].

### 2.12. Functional Enrichment Analysis

The predicted and validated downregulated targets of miR-181c-5p obtained from the miRWalk tool were subsequently used in the functional enrichment analysis. To detect the biological processes as well as the implicated signaling pathways of the obtained downregulated miR-181c-5p targets in HCC, gene ontology (GO), and Kyoto Encyclopedia of Genes and Genomes (KEGG) functional enrichment analysis was performed through Enrichr (https://maayanlab.cloud/Enrichr/) (accessed on 25 January 2022). Only <0.05 *p*-value results were c regarded as statistically significant.

### 2.13. RNA Extraction and Quantitative Real Time PCR (qRT-PCR)

Following the manufacturer’s directions, hepatic tissue specimens’ and HepG2 cells’ total RNA extraction was carried out using Trizol reagent (Qiagen, Hilden, Germany). The concentration and quality of the extracted RNA were measured using a Nanodrop 2000 spectrophotometer. The first-strand cDNA was created using the miScript II RT Kit (Qiagen, Hilden, Germany) with one μg of total RNA. The miR-181c-5p, as well as F-Box and Leucine-Rich Repeat Protein 3 (Fbxl3) expression levels, were quantified using miScript SYBR Green PCR Kit with miR-181c-5p miScript Primer Assay (Qiagen, Hilden, Germany) and gene-specific primers (Table 1), respectively. Relative expression was normalized using endogenous housekeeping control U6 small nuclear RNA (snRNA) for miR-181c-5p and GAPDH for Fbxl3 quantification. The qRT-PCR for miR-181c-5p expression level was established as follows: 95 °C for 15 min; and 40 cycles of 94 °C for 15 s, 55 °C for 30 s, and 72 °C for 30 s, while the qRT-PCR conditions for quantification of Fbxl3 were as follows: 95 °C for 15 min; and 40 cycles of 95 °C for 15 s, 60 °C for 30 s. All quantitative PCR reactions were performed on the Applied Biosystems 7500 Real-Time PCR system. A triplicate sample run was performed. The ΔΔCt method was used to determine the value of the expression fold change.

### 2.14. Statistical Analysis

The standard error (SE) was used to represent the data as mean ±. Version 21.0 of the statistical software program SPSS (SPSS Inc., Chicago, IL, USA) was carried out to perform multiple comparisons after the one-way ANOVA or Student’s *t*-test was used to identify the statistical differences. Data were approved to be statistically significant when values of *p* < 0.05. Pearson’s coefficient correlation (2-tailed) was used to investigate the correlation between miR-181c-5p and its predicted targets Fbxl3 to assess the function of miR-181c-5p.

## 3. Results

### 3.1. MiR-181c-5p Expression Data in HCC

**Figure 2:**
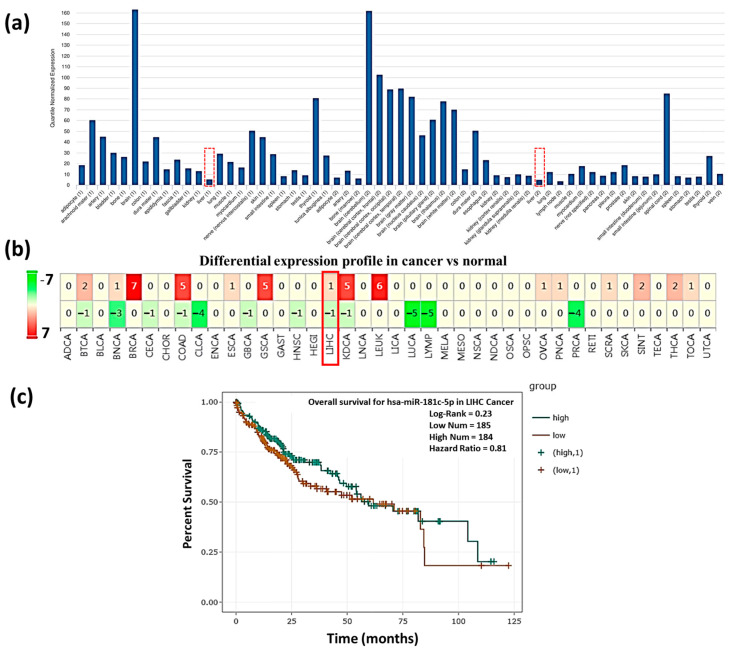
The expression status of miR-181c-5p. (**a**) The human miRNA tissue atlas demonstrated that miR-181c-5p has a low expression level in liver (red dashed box). (**b**) miR-181c-5p expression calculation using dbDEMC2 software (red box represents the expression in HCC). (**c**) Survival analysis Kaplan–Meier curves for miR-181c-5p with the survival time of HCC patients from starBase v2.0 database.

Understanding how miR-181c-5p is expressed and distributed in various tissues is important to understanding the normal development of the disease of respective tissues. To evaluate the miR-181c-5p expression in liver tissue, the human miRNA tissue atlas data were used. These results revealed that miR-181c-5p has a low expression level in the normal liver. The overall miR-181c-5p tissue-specific profile is presented in (Figure 2a). The results obtained from literature as well as databases about the miR-181c-5p expression in HCC, whether it is up- or downregulated is fluctuating, as shown in Table 2. In addition, miR-181c gives out relatively different expression statuses in multiple human malignancies, which includes HCC, as calculated using dbDEMC2 software (http://www.picb.ac.cn/dbDEMC) (accessed on 4 October 2021) (Figure 2b). Moreover, the survival analysis was obtained from the starBase v2.0 database and the Kaplan–Meier analysis curves p-value indicated that no significant difference statistically between the population survival curves for the low and high expression (Figure 2c *p* = 0.23).

### 3.2. LA-PAMAM-pmiR-181c-5p Promotes HepG2 Proliferation

**Figure 3:**
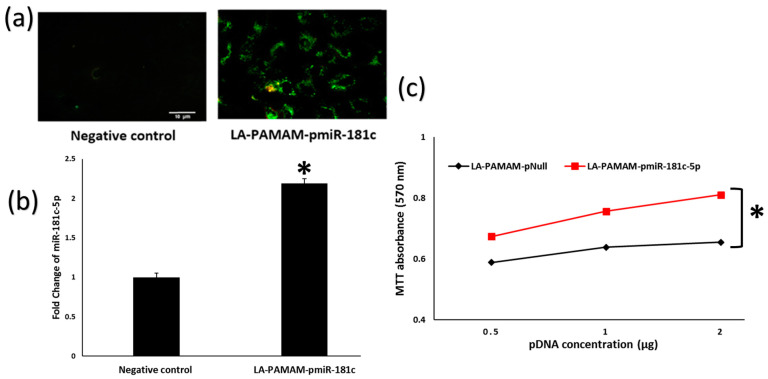
Expression of miR-181c-5p in vitro. (**a**) Green fluorescence of GFP in transfected HepG2 cells with LA-PAMAM-pmiR-181c-5p compared to untransfected HepG2 cells (Negative control). (**b**) MiR-181c-5p expression in HepG2 cells was assessed by qRT-PCR. (**c**) MTT for LA-PAMAM-pNull and LA-PAMAM-pmiR-181c-5p transfected HepG2 cells. * denotes significant *p* < 0.05 as compared to LA-PAMAM-pNull treated cells, data represented as mean ± SE.

Transfection of LA-PAMAM-pmiR-181c-5p was performed in HepG2 cells. The miR-181c-5p was successfully overexpressed after transfection, as shown by the high green fluorescence of GFP flagging the miR-181c-5p fragment in the pEGP-miR plasmid (Figure 3a). Moreover, the qRT-PCR assay was carried out in HepG2 cells and results revealed that the miR-181c-5p expression level was upregulated significantly in LA-PAMAM-pmiR-181c-5p treated cells (2.1-fold) compared to untreated cells (Negative control) (Figure 3b, *p* < 0.05). To investigate the impact of LA-PAMAM-pmiR-181c-5p on HCC cell proliferation, the transfection of HepG2 cells with different concentrations from 0.5 μg to 2 μg of LA-PAMAM-pmiR-181c-5p or LA-PAMAM-pNull as control were applied. MTT analysis indicated that miR-181c-5p significantly promoted the proliferation when the concentration of LA-PAMAM-pmiR-181c-5p is increased in HepG2 cells after 48 h of transfection, compared with the pNull transfected control groups (Figure 3c, *p* < 0.05).

### 3.3. MiR-181c-5p Overexpression Contributes to HCC Tumor Progression

**Figure 4:**
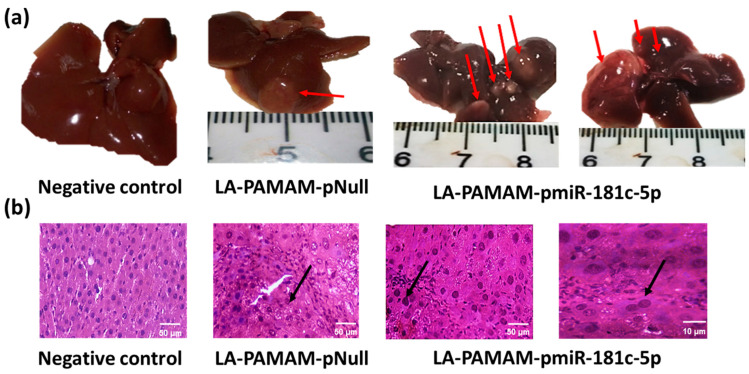
Gross morphology and microscopic features of the liver after treatment with LA-PAMAM-pmiR-181c-5p. (**a**) Macroscopic image of mice liver in all groups; red arrows in the LA-PAMAM-pNull and LA-PAMAM-pmiR-181c-5p groups represent nodules of HCC. (**b**) Histological examination of the liver tissue sections stained by H&E stain: black arrow in the LA-PAMAM-pNull and LA-PAMAM-pmiR-181c-5p groups represented a moderately differentiated HCC, which shows nuclear atypia in the shape of enlarged convoluted nuclei but retain the normal hepatocytes cytoplasmic quality. LA-PAMAM-pmiR-181c-5p represented grade 4 HCC which exhibits marked cellular pleomorphism with tumor giant cells, mitosis, and trabecular architecture absence (a feature of well-differentiated tumors).

To verify the oncogenic role of miR-181c-5p on mice with chemically developed HCC, the effect of miR-181c-5p-forced expression specifically delivered by the LA-PAMAM (LA-PAMAM-pmiR-181c-5p) was examined on the development of HCC. The liver of all groups was examined macroscopically (Figure 4a). The liver in the negative control group was dark red in color showing sharp edges, a smooth surface, and medium texture. The pmiR-181c-5p treatment showed an apparently increased number of nodules compared to pNull treatment. The LA-PAMAM-pmiR-181c-5p treated group, besides presenting a pale cirrhotic appearance with a granular surface, showed massive nodule formation more than has been apparent in the LA-PAMAM-pNull treated group. In addition, paraffin sections of hepatic tissue of all groups were histologically examined by the light microscope (Figure 4b). The negative control group showed that hepatic tissue specimens have normal architecture formed of distinct hexagonal hepatic lobules. The hepatic sinusoids appear as narrow spaces, which take the same direction as hepatic lobules. Areas of the portal tract appear at the angles of the periphery of the hepatic lobule. Hepatocytes showed central, rounded, vesicular nuclei and acidophilic granular cytoplasm and some cells are binucleated. Portal spaces were also normal, with no observed inflammatory infiltration or fatty degeneration. The liver tissue of the LA-PAMAM-pNull treated group had an appearance that is cirrhotic with a granular surface to malignant changes accompanied by a nuclear atypia, heightened nuclear:cytoplasmic ratio, and clearly visible mitotic activity. In addition, the LA-PAMAM-pNull treated group had nodules that were dysplastic and showed changes such as fatty and focal nodular hyperplasia. On the other hand, the liver of the LA-PAMAM-pmiR-181c-5p treated group exhibited malignant progression to HCC, liver lobular structure alterations, and hepatic degeneration with regenerative nodules typical of cirrhotic liver. Also, proliferative tumor lesions were present, which are accompanied with atypical mitosis as well as dysplastic aspects resembling HCCs. The LA-PAMAM-pmiR-181-5p treated liver showed different grades of HCC, ranging from dysplastic nodules, up to a well-differentiated HCC, which was significantly worse compared with that of the LA-PAMAM-pNull treated group.

### 3.4. MiR-181c-5p Overexpression Deteriorates Liver Functions in the HCC Mouse Model

**Figure 5:**
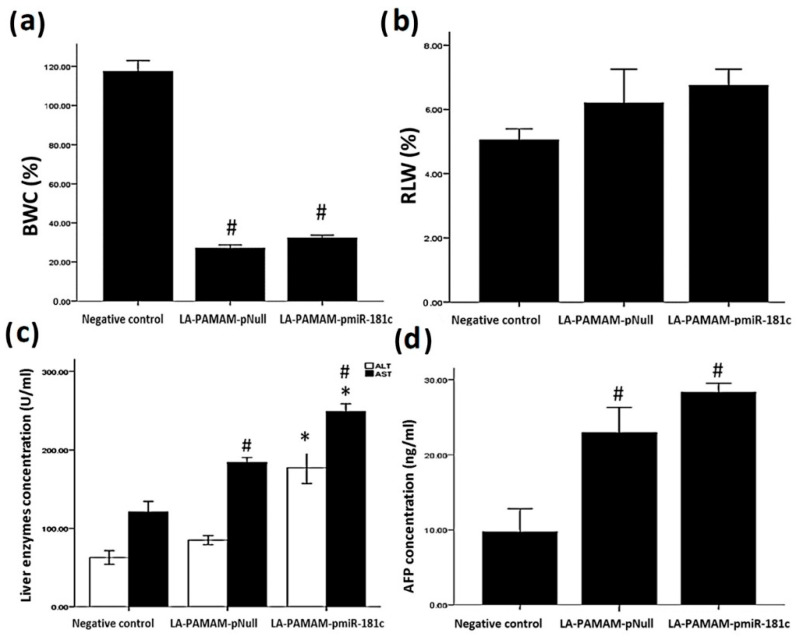
Liver functions after treatment with LA-PAMAM-pmiR-181c-5p. The effect of LA-PAMAM-pmiR-181c-5p treatment on (**a**) BWC%, (**b**) RLW, (**c**) ALT and AST activities, and (**d**) AFP between all groups. * denotes significant *p* < 0.05 as compared to LA-PAMAM-pNull treated group, # denotes significant *p* < 0.05 as compared to the negative control group. Data represented as mean ± SE.

The BWC % and RLW in all groups were examined. The weight changes showed that LA-PAMAM-pmiR-181c-5p treatment did not significantly affect the BWC % compared to the LA-PAMAM-pNull treated group; however, both groups manifested a significant decrease (*p* < 0.05) in BWC % in comparison to the negative group. In addition, there was no observed significant change in RLW among all groups (Figure 5a,b). To check the miR-181c-5p effect on liver functions, the activities of serum liver enzymes (ALT and AST), as well as serum AFP, were measured. Results demonstrate a significant increase in ALT and AST of the LA-PAMAM-pmiR-181c-5p treated group, as compared with the LA-PAMAM-pNull treated group (*p* < 0.05). Although LA-PAMAM-pmiR-181c-5p treatment did not significantly affect the AFP compared to the LA-PAMAM-pNull treated group, AFP showed a significant increase in LA-PAMAM-pmiR-181c-5p and LA-PAMAM-pNull treated groups, as compared with negative control (*p* < 0.05) (Figure 5c,d).

### 3.5. Bioinformatics Functional Analysis of MiR-181c-5p Downregulated Targets

**Figure 6:**
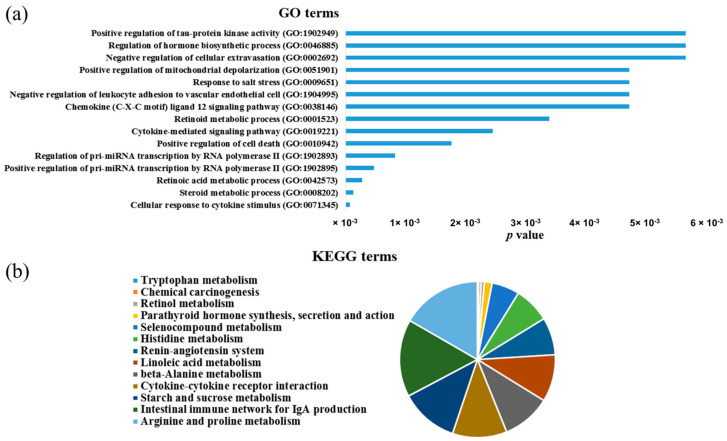
Functional enrichment analysis of downregulated miR-181c-5p targets in HCC. (**a**) Biological processes (GO) and (**b**) KEGG pathways enrichment (*p* < 0.05).

To examine the oncogenic role of the upregulated miR-181c-5p in HCC development, it was necessary to determine miR-181c-5p downregulated targets in HCC and their functions. Therefore, the targets of miR181c-5p that are mutual between the selected bioinformatics target prediction tools and the significantly downregulated genes in HCC were compiled as previously described in the Methods section. GO and KEGG terms were obtained to determine the most significantly enriched biological process and pathways of the downregulated overlapping target genes (*p* < 0.05). The results revealed that the miR-181c-5p downregulated target genes in HCC were significantly enriched in 123 biological process terms, such as cellular response to cytokine stimulus, steroid metabolic process, retinoic acid metabolic process, positive regulation of pri-miRNA transcription by RNA polymerase II, and regulation of pri-miRNA transcription by RNA polymerase II (Supplementary Table S1, Figure 6a). Enriched KEGG pathways indicated that the miR-181c-5p downregulated target genes were categorized primarily into 13 statistically significant cancer-related pathways, which are associated with HCC (Supplementary Table S1, Figure 6b). KEGG pathway analysis revealed that the downregulated genes were primarily enriched in tryptophan metabolism, retinol metabolism, chemical carcinogenesis, and parathyroid hormone synthesis, secretion, and action.

### 3.6. Fbxl3 as a Target for MiR-181c-5p Involved in HCC

**Figure 7:**
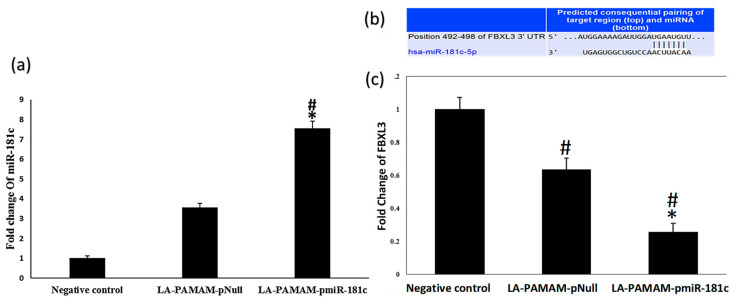
The effect of the LA-PAMAM-pmiR-181c-5p on Fbxl3 expression in vivo. (**a**) MiR-181c-5p expression in all mice groups was performed by qRT-PCR. (**b**) The miR-181c-5p binding sites on the 3 prime untranslated region (3’UTR) of Fbxl3 mRNA predicted by TargetScan. (**c**) Expression of Fbxl3 mRNA was assessed by qRT-PCR in all mice groups. * denotes significant *p* < 0.05 compared to LA-PAMAM-pNull group, # denotes significant *p* < 0.05 as compared to the negative control group. Data represented as mean ± SE.

To prove the oncogenic effect of miR-181c-5p in vivo, the qRT-PCR assay was performed. Results revealed that the miR-181c-5p expression level was significantly upregulated in the HCC tissue of the LA-PAMAM-pNull treated group (3.5-fold) compared to the negative control group (*p* < 0.05) and the miR-181c-5p expression level was significantly upregulated in HCC tissue of the LA-PAMAM-pmiR-181c-5p treated group (7.5-fold) compared to the LA-PAMAM-pNull treated group (Figure 7a *p* < 0.05). The miRWalk v2.0 manifested that “Fbxl3” is a novel predicted target for miR-181c-5p. MiRanda and TargetScan miRNA-target prediction tools were utilized to confirm this result (Figure 7b). To check the effect of the miR-181c-5p forced expression on its predicted target, the qRT-PCR analysis was carried out to assess the Fbxl3 mRNA expression levels in all mice groups as an example of miR-181c-5p potential targets significantly downregulated in HCC [52,55]. Results revealed that the LA-PAMAM-pmiR-181c-5p group showed significantly low expression (*p* < 0.05) of Fbxl3 mRNA (0.26 folds), compared to the LA-PAMAM-pNull treated group (0.64 folds) (Figure 7c). Moreover, significant downregulation of the Fbxl3 mRNA expression level in the LA-PAMAM-pmiR-181c-5p and LA-PAMAM-pNull treated groups were observed as compared with negative control (*p* < 0.05).

### 3.7. Correlation Analysis

Pearson’s coefficient correlation was performed to investigate the relationship of miR-181c-5p with Fbxl3 in the negative control, LA-PAMAM-pNull, and LA-PAMAM-pmiR-181c-5p treated mice groups. As displayed in Table 3, MiR-181c-5p was significantly and negatively correlated with Fbxl3.

## 4. Discussion

The molecular mode of the miR-181c-5p, whether to stimulate or inhibit the HCC progression is controversial and not fully elucidated yet. The miR-181c-5p expression status results obtained from the miRNA disease association databases that are mostly used in the literature; dbDEMC [44], HMDD [45], miR2Disease [46], and miRcancer [47], and survival analysis confirmed this fluctuation and provides strong bases for its involvement in HCC development. In this research, the role of miR-181c-5p in HCC progression was unraveled by its specific and targeted delivery using LA-PAMAM and forced expression in HepG2 and mouse liver with chemically induced HCC. The present results revealed a significantly elevated miR-181c-5p expression level in the HCC tissue of the LA-PAMAM-pNull treated mice group when compared to the negative control mice group. This result is in agreement with the previous research which demonstrated that mature miR-181 family members increased significantly in twenty cases of HCC, HCC stem cells, and progenitors [21,31].

In a recent study, we selectively delivered and restored miR-218 expression in HCC by constructing a biocompatible hyperbranched polyamidoamine with lactobionic acid (LA-PAMAM) decoration. The LA moieties were reported by our group to enhance the cellular uptake and efficient delivery of LA-PAMAM-pmiR-218 to HepG2 and HCC tissue in mice by targeting ASGP-Rs that are highly expressed on HCC cells. That study revealed that ASGPR expression was upregulated significantly in the tissue of HCC compared with the tissue of the normal liver and the receptor competition assay confirmed that LA-PAMAM-pmiR-218 was captured by endocytosis, mediated prevalently by ASGPR. Additionally, LA-PAMAM-pmiR-218 cellular uptake was increased compared with the naked pmiR-218 and PAMAM-pmiR-218. Moreover, the cytotoxicity of LA-PAMAM is very low in comparison to PAMAM and PEI on HepG2 cells. LA moiety conjunction could shield the cationic charges on the PAMAM polymer, which enhances its biocompatibility and reduces cytotoxicity [43]. In agreement with the previous study, transfection of LA-PAMAM-pmiR-181c-5p to HepG2 cells in the current study showed successful overexpression of pmiR-181c-5p. Here, the increased miR-181c-5p expression enhanced the viability of HepG2 cells. Furthermore, overexpression of miR-181c-5p could promote the development of HCC. Our results are in agreement with several studies that suggested that the family of miR-181, including miR-181c, could activate hepatic progenitor cells and HCC by blocking HCC cell differentiation and enhancing HCC development and progression. These roles are mediated through the Wnt/β-catenin signaling pathway [31,53]. It was revealed, on the other hand, that miR-181c could repress cell cycle, apoptosis, and metastasis in HCC through targeting oncogenic secreted phosphoprotein 1 (SPP1) [33].

The features of the liver were consistent with this fact, wherein the LA-PAMAM-pmiR-181c-5p treated group showed an increased number of HCC nodules, severe malignant histological changes, and upregulation in liver function enzymes (ALT and AST) in the serum, in correspondence with the malignant features shown by T. Uehara et al., 2014 [56], which were evident in 100% of all mice compared to the LA-PAMAM-pNull group, indicating that miR-181c-5p might be correlated with the progression of HCC.

miR-181c-5p downregulated targets in HCC were enriched significantly in different pathways and biological processes associated with HCC. The twenty miR-181c-5p targets significantly predicted in this study, “PLAC8, KBTBD11, CXCL12, FOS, MME, FBXL3, KMO, EGR1, DCN, BCHE, LIFR, CYP26A1, HSD11B1, CYP2C8, TMEM27, ITLN1, GPM6A, CNDP1, GYS2, and INMT” were downregulated in HCC [52]. For example, one of these targets is Decorin (DCN), an effective protein involved in the transforming growth factor-β (TGF-β) signaling pathway, which decreased TGF-β bioavailability [57]. In addition, DCN deficiency promoted hepatic carcinogenesis, and Decorin null (Dcn−/−) mice developed increased tumors after treatment with DEN [58]. The current analysis showed the proposed impact of miR-181c-5p to act as oncomiR and to control many significantly enriched pathways and biological processes related to HCC, such as immune system and metabolic pathways, cellular response to cytokine stimulus, cytokine–cytokine receptor interaction, and the intestinal immune network for IgA production, as well as tryptophan metabolism, retinol metabolism, arginine and proline metabolism, chemical carcinogenesis, and parathyroid hormone synthesis, secretion, and action. Involvement of these pathways in our chemically induced HCC model is highly probable because DEN is a hepatotoxic chemical and can lead to hepatocytes’ necrotic cell death, which contain pre-made interleukin (IL)-1α. This cytokine release can trigger an inflammatory chain reaction which ultimately leads to high expression of tumor necrosis factor (TNF), IL-6, and hepatocyte growth factor, which can act to start the carcinogenesis of hepatocytes [59]. Another study analyzed the chemical carcinogenesis pathway-specific process and reported HCC pathogenesis and progression through the downregulation of Hydroxysteroid 11-β Dehydrogenase 1 (HSD11B1) [60]. It has been established that the cytokine–cytokine receptor interaction pathway may be a key pathway associated with the development of HCC [61]. Additionally, activation of the intestinal immune network for the IgA production signaling pathway contributes to HCC cell proliferation and migration [62]. Further, metabolism appeared to be closely related to cancer epigenetics and altered metabolism stimulates tumor proliferation and metastasis [63]. Amino acids are vital nutrients and energy sources for tumor cells associated with lipid, glucose, and nucleotide metabolism, which are significant for the invasion, proliferation, and metastasis of tumor [64]. Tryptophan metabolism has an essential role in the HCC occurrence and development. It was revealed that the tryptophan side-chain oxidase (TSO) enzyme, which can break down tryptophan, had a restrictive effect on the HCC cell lines’ proliferation, invasion, and migration [65]. It was indicated that argininosuccinate synthase 1 (ASS1), which is a rate-limiting enzyme for arginine biosynthesis, inhibited the metastasis of HCC by suppressing the STAT3 signaling pathway [66]. An altered retinol metabolism pathway is implicated in HCC [67]. The associations between retinoids and hepatic disease have been demonstrated, involving retinoid activity loss in HCC cell lines and reduced retinoid reserves in the liver, as well as the transformed retinoid signaling in cirrhosis and HCC patients [68]. The hepatic retinoid signaling loss has been correlated with more prompt progression of the development of liver disease emerging from reactive oxygen species [69]. Acyclic retinoid (ACR) is a synthetic retinoid that is reported to suppress liver tumors induced chemically, as well as spontaneous HCC development in rodents by stimulating apoptosis and inhibiting cellular proliferation in HCC [70]. It was determined that ACR represses Ras/MAP kinase signal transduction and preserves the function of retinoid X receptor α (RXRα), which is a substantial nuclear receptor involved in the process of hepatocarcinogenesis, as its ligand represses HCC development [71]. Furthermore, it has been determined that ACR regulates the growth of HCC via repressing the expression of transforming growth factor α (TGFα) [72] through the modulation of fibroblast growth factor signaling [73] and platelet-derived growth factor signaling [74]. The level of cytochrome P450 members, such as CYP2C8 and CYP26A1, was downregulated in consistence with the progression of HCC in patients. Both CYP2C8 and CYP26A1 are involved in retinol metabolism [75].

Fbxl3 overexpression suppresses cell proliferation, stimulates cell apoptosis, arrests cell cycle, and prohibits cell invasion and migration efficiently, and has tumor inhibition prospects. In addition, Fbxl3 was reported to be downregulated in HCC [55,76,77]. A previous study demonstrated that miR-181d can act as an oncomiR that is upregulated in colorectal cancer (CRC) tissues and identified Fbxl3 as a direct target of miR-181d [78]. The miR-181c and miR-181d clusters are located similarly on chromosome 19 and share identical 8 base seed sequences [79], which serve as the main factor in the base complementation process and confirms the results of the bioinformatics analysis, which predicted Fbxl3 as a strong target for miR-181c-5p. The possible molecular effect of miR-181c-5p-forced expression on the expression level of its predicted target “Fbxl3” in HCC liver tissues was investigated and the results revealed that the miR-181c-5p expression was inversely correlated with Fbxl3 mRNA levels, which strongly suggested Fbxl3 as a possible target for miR-181c-5p in the HCC model.

## 5. Conclusions

In brief, the current study confirmed that the upregulation of miR-181c-5p is oncogenic in HCC in vitro and in vivo. As a step to identify the involved molecular mechanism, a novel correlation at which miR-181c-5p downregulates the expression of the Fbxl3 gene was proposed here. According to the miR-181c-5p biological mechanisms proposed here, it is valuable to further validate these molecular mechanisms for regulating HCC progression and to investigate their therapeutic significance. The inhibition of miR-181c-5p may represent a promising therapeutic strategy for HCC patients in the future by inhibiting all these pathways and biological processes. We are also proposing a future study on HCC patients to determine the correlation between mir-181c-5p and Fbxl3 in the diagnosis and prognosis of the HCC cases.

## Figures and Tables

**Table 1:** QRT-PCR primer sequences list.

Gene	Forward Sequence	Reverse Sequence
mGAPDH mFbxl3	5′-TCATCATCTCCGCCCCTTCT-3′ 5′-TAGACGACACCCCAGTCGAT-3′	5′-TGATGGCATGGACTGTGGTC-3′ 5′-CACAGAATGCCTGCTGGAGA-3′

**Table 2:** MiR-181c-5p different expression in HCC.

Expression Status	Design	Reference	Database
Down	Microarray on HCCs and LCs	L. Gramantieri et al. (2007) [32]	MiR2Disease Base [46]
Down	Hep3B vs L02 cells	J. Wang et al. (2019) [33]	
Down	Microarray on tumor tissues and adjacent non-tumor tissues	J. Ai et al. (2019) [34]	
Up	HpSC-HCC vs MH-HCC	J. Ji et al. (2009) [31]	MiR2Disease Base [46] MiR Cancer [47]
Up	Human HCC cell lines (Hep3B, HuH7, HuH1, MHCC97 and Smmc7721)	J. Ji et al. (2011) [53]	HMDD [45]
Up	Microarray on tumor and non-tumor hepatic tissue specimens of HCC patients	Sato F et al. (2011) [54]	dbDEMC2 [44]

LCs; Liver Cirrhosis, MH-HCC; Mature Hepatocyte-like HCC, HpSC-HCC; Hepatic Stem Cell-like HCC.

**Table 3:** Correlation between miR-181c-5p with Fbxl3 in HCC mouse model.

Gene	Fold Change miR-181c-5p	Fold Change Fbxl3
Fbxl3		
Pearson Correlation	−0.952 **	1
*p*-value	0.000	-

** Correlation is significant at the 0.01 level (2-tailed).

## Data Availability

All data generated or analyzed during this study are included in this published article (and its supplementary information files).

## Supplementary Materials

The following supporting information can be downloaded at: https://www.mdpi.com/article/10.3390/genes13122343/s1, Figure S1: Gross morphology and microscopic features of liver after DEN/CCl4 treatment; Table S1: KEGG and GO terms of downregulated miR-181c-5p targets in HCC.
