# Supplementary material for: In Silico and In Vivo Evaluation of microRNA-181c-5p’s Role in Hepatocellular Carcinoma
Source: Genes (Basel). 2022 Dec 12;13(12):2343. doi: 10.3390/genes13122343 (PMC9777864; doi:10.3390/genes13122343)
Supplement: Supplementary file 1 [file genes-13-02343-s001.zip › Figure S1.pdf]

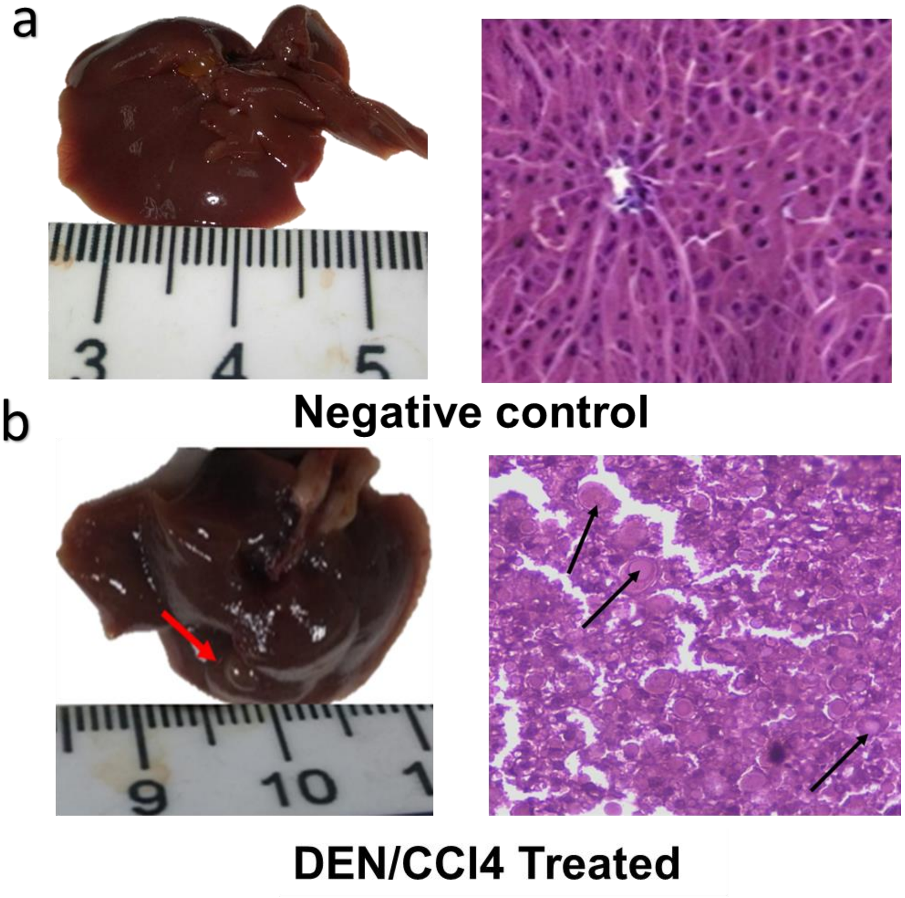


**Supplementary Figure S1. Gross morphology and microscopic features of liver after DEN/ CCl4 treatment.** Macroscopic and microscopic images of mice liver in **(a)** Negative control **(b)** DEN/CCl4 treated. The red arrow represent nodule of HCC. DEN/CCl4 treated group H&E showed sheets and nests of large tumor cells with pleomorphic nuclei, prominent nucleoli, abundant granular eosinophilic cytoplasm, scattered Mallory bodies (dense eosinophilic cytoplasmic structures), and distinct cell borders. Prominent internuclear inclusion bodies (black arrows)
