# Supplementary material for: In Silico and In Vivo Evaluation of microRNA-181c-5p’s Role in Hepatocellular Carcinoma
Source: Genes (Basel). 2022 Dec 12;13(12):2343. doi: 10.3390/genes13122343 (PMC9777864; doi:10.3390/genes13122343)
Supplement: Supplementary file 1 [file genes-13-02343-s001.zip › Table S1.pdf]

Supplementary Table S1. KEGG and GO terms of downregulated miR-181c-5p targets in HCC

KEGG terms of downregulated miR-181c-5p targets in HCC	*p* value
Tryptophan metabolism	7.20E-04
Chemical carcinogenesis	0.001417408
Retinol metabolism	0.001876299
Parathyroid hormone synthesis, secretion and action	0.004486288
Selenocompound metabolism	0.016034072
Histidine metabolism	0.020703446
Renin-angiotensin system	0.021634798
Linoleic acid metabolism	0.027205317
beta-Alanine metabolism	0.02813081
Cytokine-cytokine receptor interaction	0.03143107
Starch and sucrose metabolism	0.033666281
Intestinal immune network for IgA production	0.044647745
Arginine and proline metabolism	0.046466455

GO terms of downregulated miR-181c-5p targets in HCC	*p* value
cellular response to cytokine stimulus (GO:0071345)	7.00E-05
steroid metabolic process (GO:0008202)	1.25E-04
retinoic acid metabolic process (GO:0042573)	2.74E-04
positive regulation of pri-miRNA transcription by RNA polymerase II (GO:1902895)	4.71E-04
regulation of pri-miRNA transcription by RNA polymerase II (GO:1902893)	8.26E-04
positive regulation of cell death (GO:0010942)	0.001768751
cytokine-mediated signaling pathway (GO:0019221)	0.00245992
retinoid metabolic process (GO:0001523)	0.003401386
chemokine (C-X-C motif) ligand 12 signaling pathway (GO:0038146)	0.004741392
negative regulation of leukocyte adhesion to vascular endothelial cell (GO:1904995)	0.004741392
response to salt stress (GO:0009651)	0.004741392
positive regulation of mitochondrial depolarization (GO:0051901)	0.004741392
negative regulation of cellular extravasation (GO:0002692)	0.005687116
regulation of hormone biosynthetic process (GO:0046885)	0.005687116
positive regulation of tau-protein kinase activity (GO:1902949)	0.005687116
response to heparin (GO:0071503)	0.005687116
negative regulation of retinoic acid receptor signaling pathway (GO:0048387)	0.006631989
lipid hydroxylation (GO:0002933)	0.006631989
negative regulation of dendritic cell apoptotic process (GO:2000669)	0.006631989
regulation of ketone biosynthetic process (GO:0010566)	0.006631989
positive regulation of hormone metabolic process (GO:0032352)	0.006631989
positive regulation of membrane depolarization (GO:1904181)	0.006631989
amine metabolic process (GO:0009308)	0.007576012
regulation of actin filament length (GO:0030832)	0.007576012
benzene-containing compound metabolic process (GO:0042537)	0.007576012

integrin activation (GO:0033622)	0.007576012
monocarboxylic acid metabolic process (GO:0032787)	0.008015796
kynurenine metabolic process (GO:0070189)	0.008519186
regulation of actin filament organization (GO:0110053)	0.008519186
organic cyclic compound catabolic process (GO:1901361)	0.008519186
response to cytokine (GO:0034097)	0.008787903
negative regulation of vascular endothelial growth factor signaling pathway (GO:1900747)	0.009461511
regulation of tau-protein kinase activity (GO:1902947)	0.009461511
regulation of leukocyte tethering or rolling (GO:1903236)	0.009461511
omega-hydroxylase P450 pathway (GO:0097267)	0.009461511
indolalkylamine catabolic process (GO:0046218)	0.009461511
positive regulation of transcription by RNA polymerase II (GO:0045944)	0.009475091
glucocorticoid biosynthetic process (GO:0006704)	0.010402987
glucocorticoid metabolic process (GO:0008211)	0.010402987
regulation of mitochondrial depolarization (GO:0051900)	0.010402987
tryptophan catabolic process (GO:0006569)	0.010402987
tryptophan metabolic process (GO:0006568)	0.010402987
regulation of dendritic cell apoptotic process (GO:2000668)	0.010402987
induction of positive chemotaxis (GO:0050930)	0.010402987
fat-soluble vitamin catabolic process (GO:0042363)	0.011343616
negative regulation of cellular response to vascular endothelial growth factor stimulus (GO:1902548)	0.011343616
angiotensin maturation (GO:0002003)	0.011343616
regulation of angiotensin levels in blood (GO:0002002)	0.011343616
replicative senescence (GO:0090399)	0.011343616
dermatan sulfate biosynthetic process (GO:0030208)	0.011343616
negative regulation of leukocyte apoptotic process (GO:2000107)	0.011343616
positive regulation of calcium ion import (GO:0090280)	0.012283398
chondroitin sulfate catabolic process (GO:0030207)	0.012283398
glucan biosynthetic process (GO:0009250)	0.012283398
glycogen biosynthetic process (GO:0005978)	0.012283398
dermatan sulfate metabolic process (GO:0030205)	0.012283398
carboxylic acid biosynthetic process (GO:0046394)	0.012283398
regulation of retinoic acid receptor signaling pathway (GO:0048385)	0.013222334
steroid catabolic process (GO:0006706)	0.013222334
response to chemokine (GO:1990868)	0.013222334
positive regulation of lymphocyte migration (GO:2000403)	0.013222334
protein trimerization (GO:0070206)	0.014160425
dermatan sulfate proteoglycan biosynthetic process (GO:0050651)	0.014160425
icosanoid biosynthetic process (GO:0046456)	0.014160425
protein homotrimerization (GO:0070207)	0.014160425
regulation of intrinsic apoptotic signaling pathway in response to DNA damage (GO:1902229)	0.015097671
aromatic amino acid family catabolic process (GO:0009074)	0.015097671
positive regulation of positive chemotaxis (GO:0050927)	0.016034072
regulation of steroid biosynthetic process (GO:0050810)	0.016034072

amyloid-beta metabolic process (GO:0050435)	0.016034072
regulation of protein sumoylation (GO:0033233)	0.016969631
epoxygenase P450 pathway (GO:0019373)	0.017904347
regulation of vascular endothelial growth factor signaling pathway (GO:1900746)	0.017904347
DNA alkylation (GO:0006305)	0.017904347
DNA methylation or demethylation (GO:0044728)	0.017904347
positive regulation of mitochondrial fission (GO:0090141)	0.017904347
positive regulation of monocyte chemotaxis (GO:0090026)	0.017904347
regulation of T cell migration (GO:2000404)	0.018838221
regulation of protein modification by small protein conjugation or removal (GO:1903320)	0.018838221
chondroitin sulfate biosynthetic process (GO:0030206)	0.019771253
regulation of response to cytokine stimulus (GO:0060759)	0.020703446
exogenous drug catabolic process (GO:0042738)	0.020703446
DNA methylation (GO:0006306)	0.021634798
drug catabolic process (GO:0042737)	0.021634798
positive regulation of transcription, DNA-templated (GO:0045893)	0.023128019
regulation of calcium ion import (GO:0090279)	0.023494986
positive regulation of T cell migration (GO:2000406)	0.023494986
regulation of mitochondrial fission (GO:0090140)	0.023494986
negative regulation of intrinsic apoptotic signaling pathway in response to DNA damage (GO:1902230)	0.024423824
regulation of monocyte chemotaxis (GO:0090025)	0.024423824
positive regulation of glucose import (GO:0046326)	0.024423824
chondroitin sulfate proteoglycan biosynthetic process (GO:0050650)	0.025351824
monocarboxylic acid catabolic process (GO:0072329)	0.025351824
negative regulation of signal transduction (GO:0009968)	0.026146105
glycogen metabolic process (GO:0005977)	0.026278988
lipid catabolic process (GO:0016042)	0.027205317
regulation of actin polymerization or depolymerization (GO:0008064)	0.027205317
estrogen metabolic process (GO:0008210)	0.02813081
alpha-amino acid catabolic process (GO:1901606)	0.02813081
long-chain fatty acid biosynthetic process (GO:0042759)	0.02813081
chondroitin sulfate metabolic process (GO:0030204)	0.02813081
negative regulation of response to DNA damage stimulus (GO:2001021)	0.02905547
positive regulation of mononuclear cell migration (GO:0071677)	0.02905547
proteolysis (GO:0006508)	0.029880426
positive regulation of glucose transmembrane transport (GO:0010828)	0.029979296
negative regulation of epithelial cell migration (GO:0010633)	0.032745781
regulation of glucose import (GO:0046324)	0.03458595
positive regulation of calcium ion transport (GO:0051928)	0.03458595
positive regulation of cell development (GO:0010720)	0.035504791
negative regulation of endothelial cell migration (GO:0010596)	0.035504791
cellular response to oxygen-containing compound (GO:1901701)	0.037104908
stem cell differentiation (GO:0048863)	0.037339987
sulfur compound catabolic process (GO:0044273)	0.037339987

regulation of chemokine production (GO:0032642)	0.039171875
lipid modification (GO:0030258)	0.04008658
alpha-amino acid metabolic process (GO:1901605)	0.042825748
regulation of cellular metabolic process (GO:0031323)	0.043737157
regulation of protein metabolic process (GO:0051246)	0.044647745
response to glucose (GO:0009749)	0.04555751
regulation of cellular protein metabolic process (GO:0032268)	0.04555751
blood circulation (GO:0008015)	0.04737458
plasma membrane bounded cell projection morphogenesis (GO:0120039)	0.048281886
positive regulation of chemokine production (GO:0032722)	0.049188373
positive regulation of macroautophagy (GO:0016239)	0.049188373
